# Supplementary figures and images for: Genetic Influences on Translation in Yeast
Source: PLoS Genet. 2014 Oct 23;10(10):e1004692. doi: 10.1371/journal.pgen.1004692 (PMC4207643; doi:10.1371/journal.pgen.1004692)

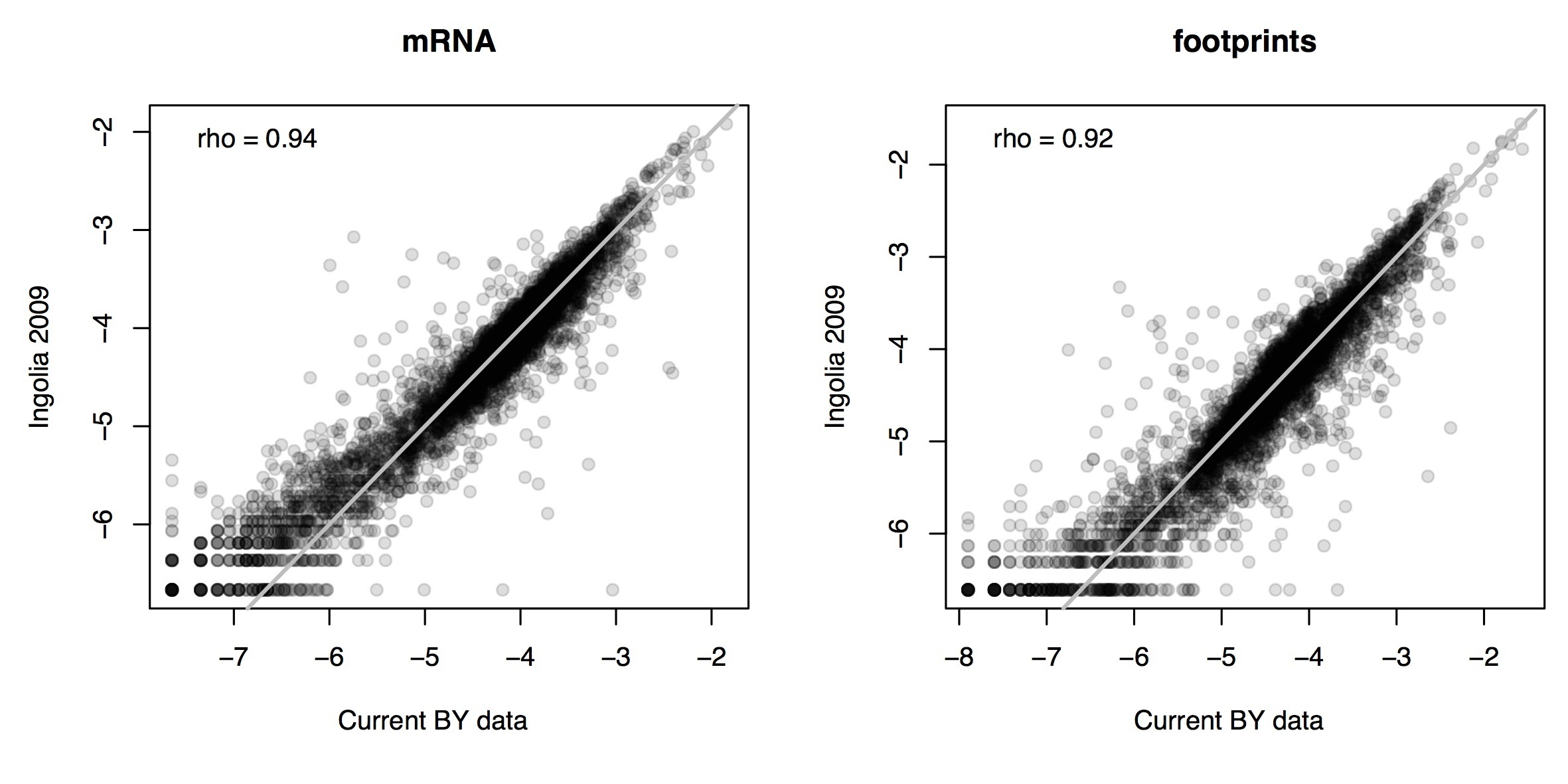

Supplement: Figure S1 — Comparison to Ingolia et al. 2009 data. Shown are log10 transformed normalized read counts. The grey line marks identity. (JPG) [file pgen.1004692.s001.jpg]

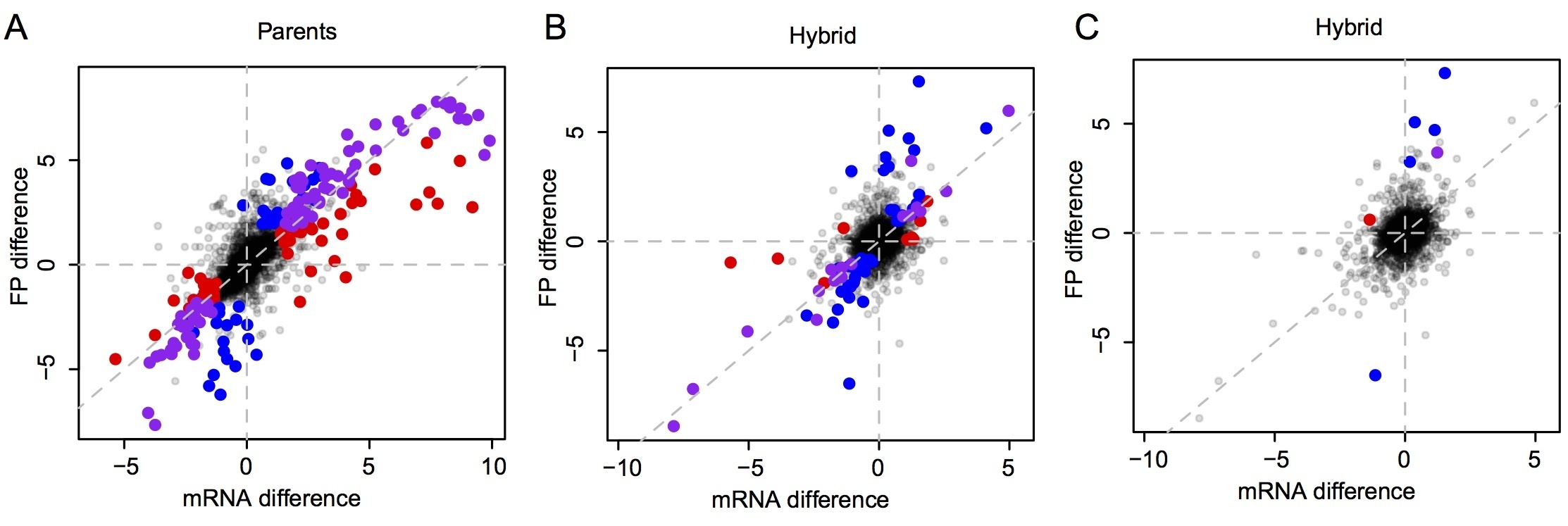

Supplement: Figure S2 — mRNA vs. footprint differences identified by DESeq. Shown are log2-transformed fold changes. A: parents, B & C: hybrid ASE. A & B: genes with a significant (Benjamini-Hochberg corrected p<0.05) mRNA (red), footprint (blue) or both mRNA and footprint (purple) difference. C: genes with a significant TE difference. Red: genes with only a significant mRNA difference, blue: genes with only a significant footprint difference, purple: genes with both a significant mRNA and footprint difference. (JPG) [file pgen.1004692.s002.jpg]

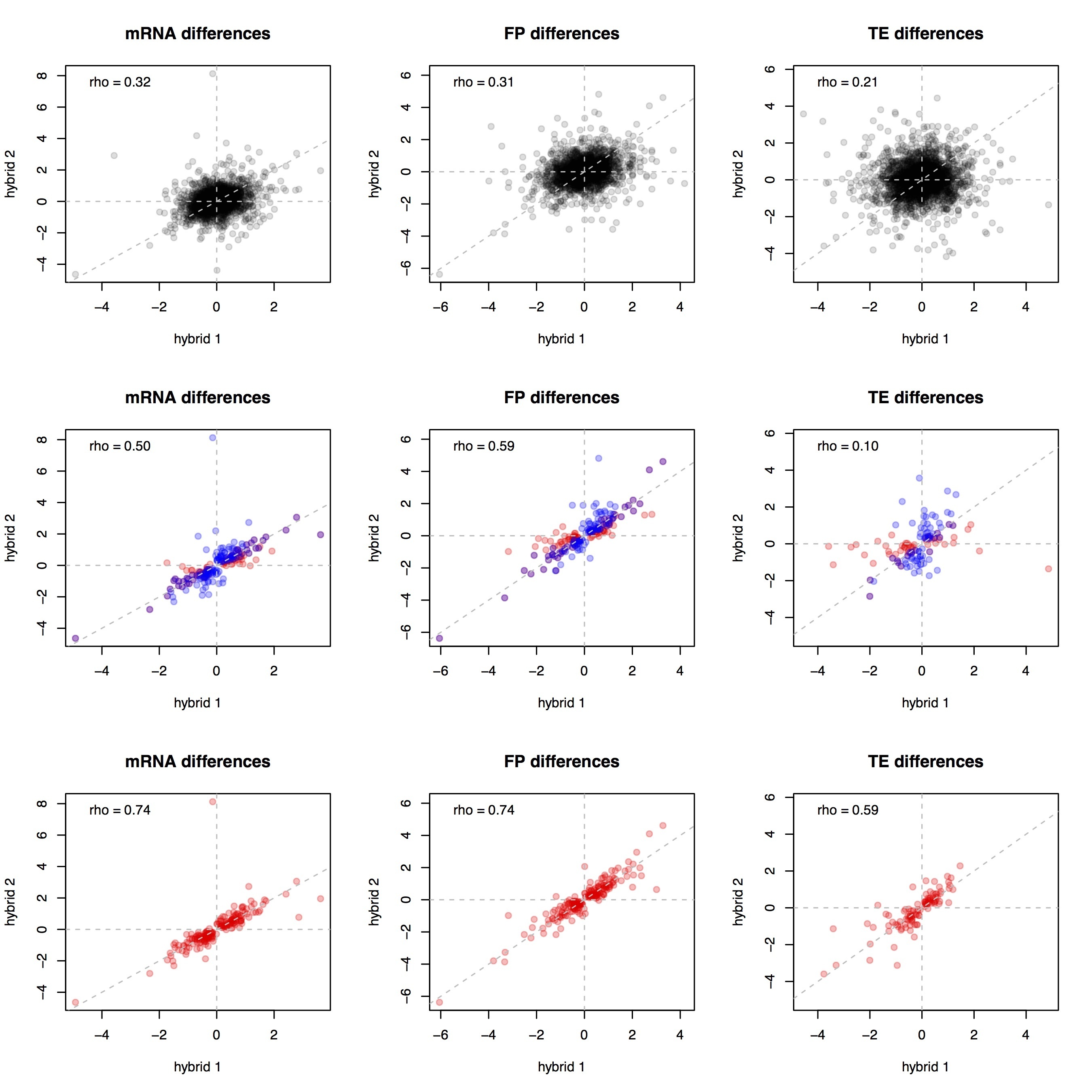

Supplement: Figure S3 — Reproducibility of hybrid measurements. Shown are log2-transformed fold changes. Grey diagonals mark identity. Top row: all genes. Middle row: significant genes in one and/or the other replicate. Bottom row: significant genes in the combined hybrid data. Spearman correlation coefficients between replicates are given in each panel. (JPG) [file pgen.1004692.s003.jpg]

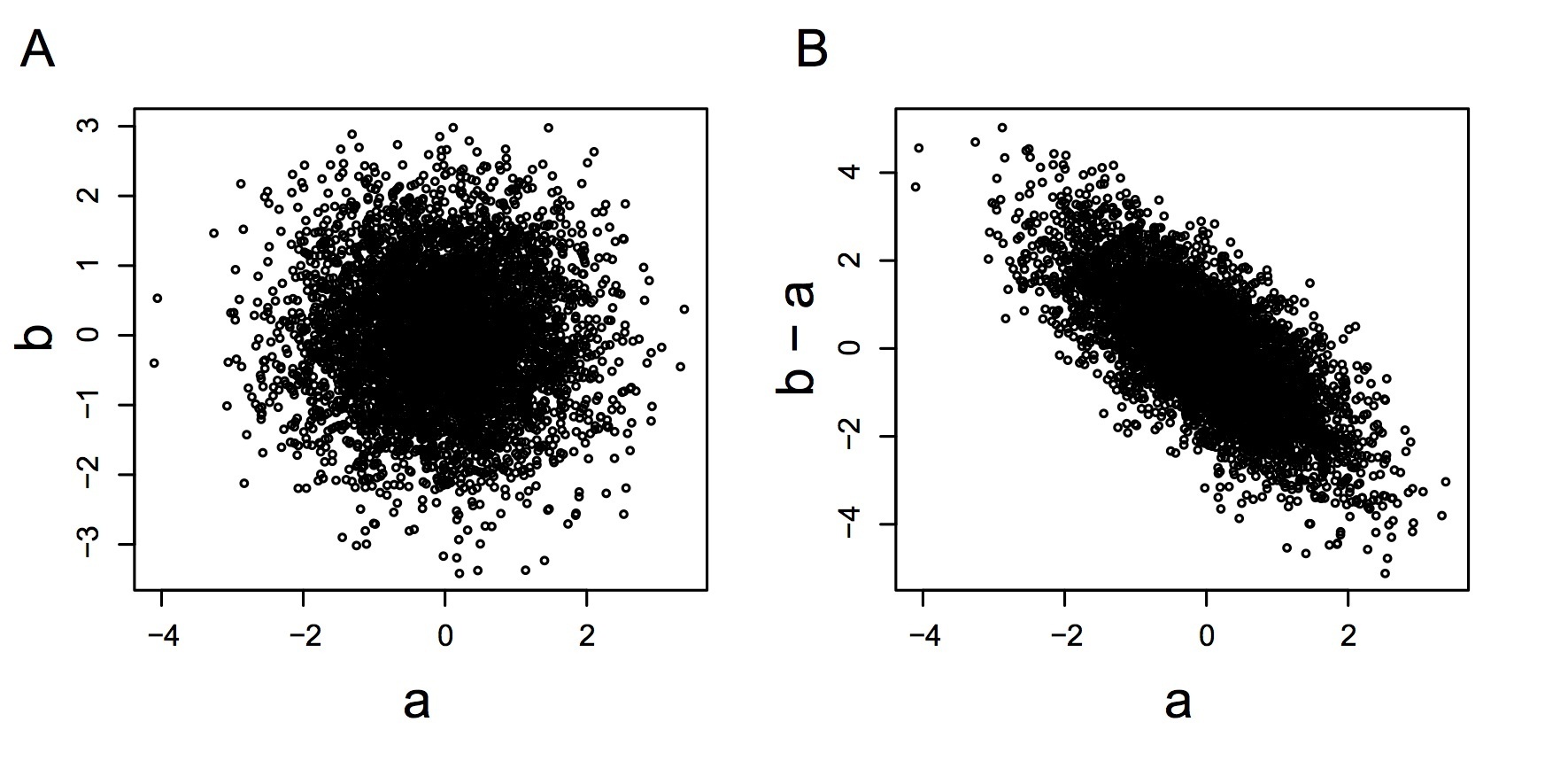

Supplement: Figure S4 — Spurious correlations induced by correlations between a log ratio and its denominator. A. A scatterplot of two random samples a and b of size 5,000 from a standard normal distribution with mean = 0 and standard deviation = 1. Note that a and b are entirely uncorrelated. B. The correlation between the quantity b - a and a is negative and highly significant because of regression to the mean. For example, when a happens to be large by chance, the corresponding value of b will usually be closer to the mean than a because it is unlikely that a large value is sampled two times by chance. Therefore, the quantity b - a is systematically more likely to be less than zero for a>0. If a and b are interpreted as the logarithms of mRNA and footprint differences, b - a is equivalent to the corresponding TE differences. A negative correlation between TE differences and mRNA differences is thus not by itself sufficient to infer translational buffering. (JPG) [file pgen.1004692.s004.jpg]

A

McManus Hybrid

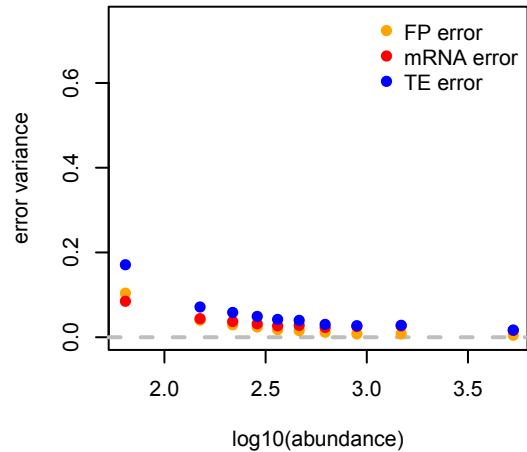

B

Artieri Hybrid

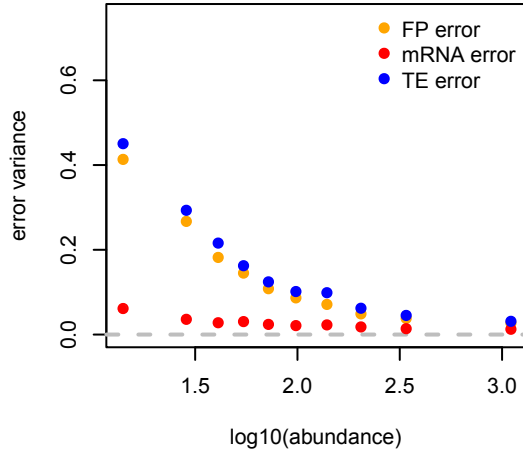

C

BY / RM Hybrid

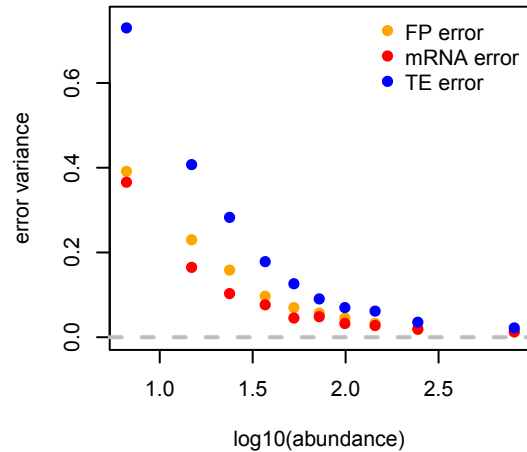

Supplement: Figure S5 — Replicate noise in different hybrid datasets. For each gene in each dataset, we calculated the log2 fold change between the alleles in the hybrid for mRNA, footprint, and TE separately for each of the two replicate datasets. The genes in each dataset were divided into 10 bins of increasing mRNA expression level. Within each bin, the average variance in the log2 fold change that is due to measurement error was calculated using the meas.est() function in the R smatr package [33] and plotted as a function of the mean abundance of the genes in the given bin. A & B: data from the published interspecies hybrid comparisons in McManus et al. [34] (A) and Artieri & Fraser [35] (B). C: Data from the BY/RM hybrid. In all datatsets, error is higher for genes with lower abundance. Footprints typically have higher error than mRNA. However, the degree to which these two data types differ varies between datasets (e.g. compare A to B). The error variance in TE is the sum of the errors in mRNA and footprints. (PDF) [file pgen.1004692.s005.pdf]
