# Supplementary material for: Genetic Influences on Translation in Yeast
Source: PLoS Genet. 2014 Oct 23;10(10):e1004692. doi: 10.1371/journal.pgen.1004692 (PMC4207643; doi:10.1371/journal.pgen.1004692)
Supplement: Table S1 — Sequencing and alignment statistics. Numbers are given in millions of reads. 1A version of the BY reference genome with all known single nucleotide differences set to the RM allele. (DOCX) [file pgen.1004692.s006.docx]

Supplementary Table S1 – Sequencing and alignment statistics

| Strain | Data type | Raw reads | Parent comparison:  unique alignments | | ASE analyses:  unique & no mismatch | | ASE analyses:  Unique, no mismatch & spans a SNP | |
| --- | --- | --- | --- | --- | --- | --- | --- | --- |
| Reference genome |  |  | BY | Edited BY^1^ | BY | RM | BY | RM |
| BY parent | Footprint | 189 | 82 | - | 74 | - | 3.7 | - |
| BY parent | mRNA | 146 | 53 | - | 46 | - | 2.4 | - |
| RM parent | Footprint | 222 | - | 151 | - | 129 | - | 6.7 |
| RM parent | mRNA | 129 | - | 52 | - | 46 | - | 2.5 |
| BY/RM diploid 1 | Footprint | 103 | - | - | 33 | 32 | 0.9 | 0.9 |
| BY/RM diploid 1 | mRNA | 98 | - | - | 27 | 26 | 0.7 | 0.7 |
| BY/RM diploid 2 | Footprint | 108 | - | - | 44 | 42 | 1.2 | 1.2 |
| BY/RM diploid 2 | mRNA | 113 | - | - | 28 | 28 | 0.8 | 0.8 |

Numbers are given in millions of reads. ^1^A version of the BY reference genome with all known single nucleotide differences set to the RM allele.
