# Supplementary material for: Genetic Influences on Translation in Yeast
Source: PLoS Genet. 2014 Oct 23;10(10):e1004692. doi: 10.1371/journal.pgen.1004692 (PMC4207643; doi:10.1371/journal.pgen.1004692)
Supplement: Table S2 — Genomic sources of mRNA and footprint reads in the BY parent. Percentages can sum to more than 100 due to overlapping annotations, 1percent of all UTRs. (DOCX) [file pgen.1004692.s007.docx]

Supplementary Table S2 – Genomic sources of mRNA and footprint reads in the BY parent

|  | mRNA | | Ribosomal footprints | |
| --- | --- | --- | --- | --- |
|  | unique | repetitive | unique | repetitive |
| CDS | 44.4M (84%) | 5.8M (6.6%) | 79.2M (97%) | 12.2M (12%) |
| UTRs^1^ | 8.9M (17%) | 66k (0.1%) | 4.7M (5.7%) | 145k (0.1%) |
| 5’UTR | 3.1M (35%)^1^ | 34k (52%)^1^ | 3.2M (68%)^1^ | 69k (48%)^1^ |
| 3’UTR | 5.9M (66%)^1^ | 32k (48%)^1^ | 1.5M (32%)^1^ | 75k (52%)^1^ |
| rRNA | 246k (0.5%) | 79M (90%) | 950k (1.2%) | 88M (85%) |
| tRNA | 73k (0.1%) | 466k (0.5%) | 228k (0.3%) | 1.2M (1.2%) |
| Other noncoding: snoRNA, snRNA, ncRNA | 468k (0.9%) | 727 | 217k (0.3%) | 264 |
| Total | 53M | 88M | 82M | 103M |

Percentages can sum to more than 100 due to overlapping annotations

^1^percent of all UTRs
