# Supplementary material for: Genetic Influences on Translation in Yeast
Source: PLoS Genet. 2014 Oct 23;10(10):e1004692. doi: 10.1371/journal.pgen.1004692 (PMC4207643; doi:10.1371/journal.pgen.1004692)
Supplement: Table S3 — FDR-based differential expression statistics, 1q-value<0.05. (DOCX) [file pgen.1004692.s008.docx]

Supplementary Table S3 – FDR-based differential expression statistics

| Comparison | Reads | Data | Analyzed genes | 2-fold | Binomial test^1^ | Intersect |
| --- | --- | --- | --- | --- | --- | --- |
| Parent | All | mRNA | 5,316 | 331 | 4,575 | 331 (6%) |
| Parent | All | Footprint | 5,316 | 514 | 4,669 | 512 (10%) |
| Parent | All | TE | 5,316 | 135 | 4,256 | 135 (3%) |
| Parent | SNP | mRNA | 3,342 | 249 | 1,159 | 225 (7%) |
| Parent | SNP | Footprint | 3,342 | 475 | 1,486 | 441 (13%) |
| Parent | SNP | TE | 3,342 | 329 | 1,155 | 278 (8%) |
| Hybrid | SNP | mRNA | 3,342 | 100 | 529 | 65 (2%) |
| Hybrid | SNP | Footprint | 3,342 | 194 | 617 | 128 (4%) |
| Hybrid | SNP | TE | 3,342 | 216 | 638 | 148 (4%) |

^1^q-value < 0.05
