# Supplementary material for: Genetic Influences on Translation in Yeast
Source: PLoS Genet. 2014 Oct 23;10(10):e1004692. doi: 10.1371/journal.pgen.1004692 (PMC4207643; doi:10.1371/journal.pgen.1004692)
Supplement: Table S4 — Strong cis effects on translation identified by DESeq. 1Infinite fold changes indicate that there were zero counts in one of the groups. Such genes were excluded from the binomial tests reported in the main text. Genes not identified by the binomial test all had counts below the inclusion criteria for binomial testing. (1) “Dubious” ORF, footprint data shows translated region only partially overlaps with annotation. The TE difference is due to a nonsense SNP in BY that results in early termination compared to RM. (2) Based on the parental read data, YJL108C forms one ORF in RM with its upstream neighbor YJL107C. The combined ORF in RM is interrupted by a stop mutation in BY, resulting in two separate gene annotations. (3) Putative protein with frameshift in RM that leads to premature termination. Note that “dubious” ORFs were not included in our analyses of nonsense SNPs so that YDR133C and YJR015W were not included in those analyses. (4) Similar to (1), and uncharacterized ORF that in RM forms one ORF with the upstream “uncharacterized” YNR066C. (DOCX) [file pgen.1004692.s009.docx]

Supplementary Table S4 – Strong *cis* effects on translation identified by DESeq

|  | TE | mRNA |  | FP |  | Identified by binomial test? | Notes |
| --- | --- | --- | --- | --- | --- | --- | --- |
|  | p-value | Log2(fold change) | p-value | log2(fold change) | p-value |  |  |
| YBR012C | 2.6E-06 | 1.12 | 1.4E-03 | 4.69 | 3.0E-07 |  |  |
| YDL231C (*BRE4*) | 1.6E-05 | 0.16 | 0.6 | 3.23 | 3.1E-06 | Yes |  |
| YDR133C | 9.9E-07 | 1.22 | 1.8E-06 | 3.66 | 1.8E-15 | Yes | (1) |
| YJL108C (*PRM10*) | 3.7E-05 | 0.35 | 0.5 | 5.05 | 1.2E-05 |  | (2) |
| YJR015W | 1.2E-11 | -1.16 | 3.1E-03 | -6.53 | 5.1E-23 | Yes | (3) |
| YJR072C (*NPA3*) | 2.3E-08 | -1.38 | 1.9E-06 | 0.59 | 0.03 | Yes |  |
| YNL020C (*ARK1*) | 7.5E-11 | -0.69 | 0.1 | -Inf^1^ | 1.5E-15 |  |  |
| YNR065C | 8.3E-05 | 0.92 | 0.06 | Inf^1^ | 2.3E-03 |  | (4) |
| YPR192W (*AQI1*) | 2.9E-06 | 1.51 | 0.2 | 7.30 | 6.1E-23 |  |  |

^1^Infinite fold changes indicate that there were zero counts in one of the groups. Such genes were excluded from the binomial tests reported in the main text. Genes not identified by the binomial test all had counts below the inclusion criteria for binomial testing. (1) “Dubious” ORF, footprint data shows translated region only partially overlaps with annotation. The TE difference is due to a nonsense SNP in BY that results in early termination compared to RM. (2) Based on the parental read data, YJL108C forms one ORF in RM with its upstream neighbor YJL107C. The combined ORF in RM is interrupted by a stop mutation in BY, resulting in two separate gene annotations. (3) Putative protein with frameshift in RM that leads to premature termination. Note that “dubious” ORFs were not included in our analyses of nonsense SNPs so that YDR133C and YJR015W were not included in those analyses. (4) Similar to (1), and uncharacterized ORF that in RM forms one ORF with the upstream “uncharacterized” YNR066C.
