# Supplementary material for: Genetic Influences on Translation in Yeast
Source: PLoS Genet. 2014 Oct 23;10(10):e1004692. doi: 10.1371/journal.pgen.1004692 (PMC4207643; doi:10.1371/journal.pgen.1004692)
Supplement: Table S5 — Effects of translation in TE genes derived from alternative significance criteria. FDR: genes with q-values<0.05 in the binomial tests. (DOCX) [file pgen.1004692.s010.docx]

Supplementary Table S5 – Effects of translation in TE genes derived from alternative significance criteria

| Significant difference | Direction of differences | Magnitude of differences | Parent FDR | Hybrid FDR | Hybrid DESeq |
| --- | --- | --- | --- | --- | --- |
| mRNA and footprint | same | Footprint > mRNA | 1,638 (38%) | 40 (6%) | 1 |
| Footprint only | – | – | 497 (12%) | 193 (30%) | 6 |
| mRNA and footprint | same | mRNA > footprint | 904 (21%) | 20 (3%) | 0 |
| mRNA only | – | – | 401 (9%) | 147 (23%) | 1 |
| mRNA and footprint | opposite | – | 778 (18%) | 21 (3%) | 0 |
| neither | – | – | 38 (1%) | 217 (34%) | 1 |
| Sum | – | – | 4,256 | 638 | 9 |

FDR: genes with q-values < 0.05 in the binomial tests
