# Supplementary material for: Genetic Influences on Translation in Yeast
Source: PLoS Genet. 2014 Oct 23;10(10):e1004692. doi: 10.1371/journal.pgen.1004692 (PMC4207643; doi:10.1371/journal.pgen.1004692)
Supplement: Table S6 — Effects of translation in TE genes in published interspecies comparisons. (DOCX) [file pgen.1004692.s011.docx]

Supplementary Table S6 – Effects of translation in TE genes in published interspecies comparisons

| Significant difference | Direction of differences | Magnitude of differences | McManus  Parent | McManus  Hybrid | Artieri  Parent | Artieri  Hybrid |
| --- | --- | --- | --- | --- | --- | --- |
| Footprint only | – | – | 443 | 471 | 22 | 132 |
| mRNA and footprint | same | Footprint > mRNA | 552 | 249 | 669 | 287 |
| mRNA and footprint | same | mRNA > footprint | 794 | 319 | 307 | 66 |
| mRNA only | – | – | 1,001 | 778 | 120 | 229 |
| mRNA and footprint | opposite | – | 258 | 108 | 293 | 159 |
| neither | – | – | 357 | 567 | 4 | 15 |
| Sum | – | – | 3,405 | 2,492 | 1,415 | 888 |
