# Supplementary material for: Genetic Influences on Translation in Yeast
Source: PLoS Genet. 2014 Oct 23;10(10):e1004692. doi: 10.1371/journal.pgen.1004692 (PMC4207643; doi:10.1371/journal.pgen.1004692)
Supplement: Note S1 — Results and discussion of read data. (PDF) [file pgen.1004692.s014.pdf]

# Supplementary Note 1 – Genomic sources of mRNA and footprint fragments

We generated 189 million (M) and 222 M ribosomal footprint reads from BY and RM, respectively (see Supplementary Table S1 for read and alignment statistics for all samples). In parallel, we gathered mRNA data from the same yeast cultures. Of the reads, 82 M (43%) for BY and 151 M (68%) for RM mapped to unique positions in the genome. The difference in the percentage of uniquely mapping reads is likely due to differences in the efficacy of ribosomal RNA (rRNA) depletion during library construction. rRNA is transcribed from a highly repetitive region of the genome (s. below) and greatly outnumbers mRNA in exponentially growing yeast cells [1] so that even minor differences in the efficacy of rRNA depletion can lead to large differences in rRNA retention. As a consequence, more uniquely mapping mRNA sequencing reads are available in samples with more effective rRNA depletion.

We determined which genomic features were the source of the footprint and mRNA reads. These analyses were conducted on the data from the BY strain, in order to allow us to directly use the reference genome annotation (Supplementary Table S2). Of uniquely mapping mRNA reads, the vast majority (84%) corresponded to either protein coding sequences (CDS) or untranslated regions (UTRs; 17%, the sum can be more than 100% because of overlapping annotations). For footprints, 97% of uniquely mapping reads aligned to CDS, while 6% mapped to UTRs. The higher fraction mapping to CDS in footprints fits the expectation that translating ribosomes should be preferentially found on coding regions, rather than on UTRs. Notably, of mRNA reads that uniquely mapped to UTRs, 35% mapped to 5' UTRs, while this fraction was nearly twice as high (68%) in the footprints. A higher density of ribosomes in 5'UTRs than expected based on mRNA abundance may be due to ribosomes that translate upstream open reading frames (uORFs) on 5'UTRs but not 3'UTRs [2,3]. Of the reads that mapped to multiple locations in the genome, many fewer mapped to CDS and UTRs (Supplementary Table S2). These "repeat reads" were heavily dominated by the ribosomal rRNA genes: 90% of mRNA and 85% of footprint reads mapped there. The rRNA genes are each represented by a small number of gene annotations in the yeast reference genome sequence (two

annotations for 35S pre-rRNA and six annotations for different variants of 5S rRNA) which represent 100-200 tandem repeats of the rDNA locus [4]. The large number of rRNA reads therefore reflects rRNA transcription across all rDNA repeats and is in line with the high amounts of rRNAs in exponentially growing yeast [1]. In this work, we considered only reads with unique alignments.

## References for Supplementary Note S1


1. Warner JR (1999) The economics of ribosome biosynthesis in yeast. Trends in Biochemical Sciences 24: 437–440. doi:10.1016/S0968-0004(99)01460-7.

2. Ingolia NT, Ghaemmaghami S, Newman JRS, Weissman JS (2009) Genome-Wide Analysis in Vivo of Translation with Nucleotide Resolution Using Ribosome Profiling. Science (New York, NY) 324: 218–223. doi:10.1126/science.1168978.

3. Brar GA, Yassour M, Friedman N, Regev A, Ingolia NT, et al. (2012) High-Resolution View of the Yeast Meiotic Program Revealed by Ribosome Profiling. Science (New York, NY) 335: 552–557. doi:10.1126/science.1215110.

4. Venema J, Tollervey D (1999) Ribosome synthesis in Saccharomyces cerevisiae. Annual Review of Genetics 33: 261–311.
