# Supplementary material for: Genetic Influences on Translation in Yeast
Source: PLoS Genet. 2014 Oct 23;10(10):e1004692. doi: 10.1371/journal.pgen.1004692 (PMC4207643; doi:10.1371/journal.pgen.1004692)
Supplement: Note S2 — Results obtained using the DESeq significance testing framework. (PDF) [file pgen.1004692.s015.pdf]

Supplementary Note 2 – Analyses based on the DESeq framework

The DESeq analysis framework employs a negative binomial distribution to identify genes with significant expression differences while explicitly accounting for differences in the total number of reads in each sample and differences in expression noise for genes with different expression levels [1].

In the parent strains, DESeq identified 189 genes with significant (5% FDR) mRNA differences, and 145 significant footprint differences (Table 1). These numbers are substantially lower than those obtained using the binomial test due to that fact that in data without replicates, DESeq estimates noise from the differences between the two samples. Because true strain differences are conflated with technical and biological variation, this procedure overestimates the noise, resulting in a conservative test. Nevertheless, the agreement between the mRNA and footprint differences identified by DESeq was very high: of the 189 genes with an mRNA difference, 101 also had a significant footprint difference (Fisher's exact test $p < 2.2e-16$, odds ratio = 157). The direction of effect agreed for 97% (227 / 233) genes with either a significant mRNA and / or a significant footprint difference (Supplementary Figure S2A). It is not possible to calculate significant differences in TE using DESeq without biological replicates.

In the hybrid data, DESeq called 40 genes as having significant (5% FDR) allele-biased mRNA expression and 70 genes with significant allele-biased footprint counts. These numbers are again conservative compared to the binomial test. However, the difference in the numbers of significant genes between DESeq and the binomial test is smaller for the hybrid than the parental comparison. This is because for the hybrid data, DESeq uses the two biological replicates, which adds power to the test. Again, the mRNA and footprint differences in the hybrid were very similar: of the 40 genes with an mRNA difference, 26 also had a footprint difference (FET $p < 2.2e-16$, odds ratio = 166). The direction of allelic expression bias agreed for 98% (82 / 84) of genes with an mRNA and / or a footprint difference (Supplementary Figure S2B).

DESeq identified 9 genes with significant TE differences (Supplementary Table S4 & Supplementary Figure S2B). Four of these were also found by the binomial test,

and the remaining five genes had been excluded from analysis with the binomial test due to low expression. The five genes only identified by DESeq contained two additional cases where the TE difference is due to a longer ORF in RM is broken up into two annotated ORFs in BY (Supplementary Table S4).

We portioned these 9 TE genes to ask if reinforcing or buffering interactions predominate (Supplementary Table S5). There were 6 genes with a footprint but no mRNA difference, 1 gene with only an mRNA difference, 1 gene with both an mRNA and a footprint difference where the footprint difference was larger than the mRNA difference, and one gene where neither mRNA nor footprints were significantly different between alleles. Thus, there is one TE gene consistent with buffering and 7 consistent with footprints reinforcing or generating an expression difference ($\chi^2 = 4.5$, $p = 0.03$).

In sum, DESeq identified substantially fewer genes with significant differences, as expected for a conservative method [2] in an experiment with few replicates. However, the important patterns presented in the main text held using these much smaller sets of genes. Significant mRNA and footprint differences agreed very well in both parents and the hybrid. Further, among the few genes flagged as having significant differences in TE in the hybrid, there was an excess of genes with larger footprint than mRNA differences.

References for Supplementary Note S2

1. Anders S, Huber W (2010) Differential expression analysis for sequence count data. Genome Biol 11: R106. doi:10.1186/gb-2010-11-10-r106.

2. Soneson C, Delorenzi M (2013) A comparison of methods for differential expression analysis of RNA-seq data. BMC Bioinformatics 14: 91. doi:10.1101/gr.101204.109.
