# Supplementary material for: Genetic Influences on Translation in Yeast
Source: PLoS Genet. 2014 Oct 23;10(10):e1004692. doi: 10.1371/journal.pgen.1004692 (PMC4207643; doi:10.1371/journal.pgen.1004692)
Supplement: Note S3 — Details on directional effects of translation differences within and between yeast species. (PDF) [file pgen.1004692.s016.pdf]

Supplementary Note S3 – Directional effects of translation differences within and between yeast species

To test whether there is a directional excess of translational effects, we divided up the TE genes (i.e. genes where the footprint difference was significantly different from the mRNA difference) as shown in Figure 5 and Table 3. There are several choices that can be made in making these comparisons.

For example, the authors of the two comparisons of the *S. cerevisiae* and *S. paradoxus* yeast species [1,2] examined the subset of TE genes that also had an mRNA difference. Within this subset, they compared the number of reinforced genes to the sum of the buffered, completely buffered and inverted genes, i.e. all cases where the translation difference opposes the mRNA difference ("opposing" genes). These analyses leave out "FP only" genes, although "FP only" genes and reinforced genes both increase the footprint difference relative to the mRNA difference (we call these two groups "increasing" genes). Further, it is not necessarily obvious that inverted genes should be grouped with the two "buffered" categories, because the absolute magnitude of the resulting difference in protein synthesis need not be smaller than the mRNA difference. Indeed, McManus et al. only included inverted genes in their set of opposing genes if the absolute footprint difference was smaller than the absolute mRNA difference [1].

We systematically conducted all possible comparisons between different definitions of opposing and increasing genes (Figure 5 & Tables 3 & 4). Between the BY and RM parents there were more TE genes where translation increased rather than decreased or inverted the footprint difference relative to the mRNA difference, but this effect was dependent on the inclusion of the "FP only" genes in the "increased" genes. The result further depended on the precise significance cutoff used to group the TE genes (Table 4 & Supplementary Table S5).

In the BY / RM hybrid data, there were more TE genes where translation increased the footprint difference relative to the mRNA difference, but only when the "FP only" genes were included in the analyses, and only when ASE TE genes were defined using the more liberal FDR criterion (Table 4).

Given that the BY / RM result depended on the precise way in which the TE genes are grouped and compared, we conduced the same analyses for the two interspecies comparisons [1,2]. As shown in the main text, the results from [1] were robust to the precise comparison and always showed an excess of opposing effects of translation in both the parent and the hybrid comparison.

In the hybrid data from [2], we saw the same effect when conducting the comparison exactly as described by the authors (s. above). However, when "FP only" genes are included in the comparison and inverted genes are excluded, the remaining opposing genes are in the minority compared to the increasing genes (Table 4). The other two possible comparisons show no significant preference for or against the respective sets of opposing or increasing genes. In the parent data from [2], there is a significant excess of increasing genes when inverted genes are excluded from the opposing genes. When the inverted genes are included, the number of increasing and opposing effects are not statistically different (Table 4).

The two published reports of predominant opposing effects of translation between yeast species were respectively based on additional analyses. McManus et al. reported that TE differences showed a negative correlation with mRNA differences [1]. The same pattern is visible (although it was not highlighted as such) in the data by Artieri & Fraser (Figure 2A top panel in [2]) as well as in our own data (not shown). However, the TE difference is the ratio of the footprint difference and the mRNA difference. Comparisons between ratios and their components can induce "spurious" correlations [3,4] (Supplementary Figure S4). A negative correlation between TE differences and mRNA differences therefore does not provide evidence for translational buffering by itself. An excess of opposing effects in Artieri & Fraser [2] was further supported by the observation that the slope of the regression of footprint differences on mRNA differences was less than one. An alternative explanation for this slope estimate may be regression to the mean ([5] p. 58). In the presence of measurement noise, and when two observations are on similar scales (as is the case for mRNA and footprint differences), regression slopes are less than one.

References for Supplementary Note S3

1. McManus CJ, May GE, Spealman P, Shteyman A (2014) Ribosome profiling reveals post-transcriptional buffering of divergent gene expression in yeast. Genome Research 24: 422–430. doi:10.1101/gr.164996.113.

2. Artieri CG, Fraser HB (2014) Evolution at two levels of gene expression in yeast. Genome Research 24: 411–421. doi:10.1101/gr.165522.113.

3. Pearson K (1897) Mathematical Contributions to the Theory of Evolution.--On a Form of Spurious Correlation Which May Arise When Indices Are Used in the Measurement of Organs. Proceedings of the Royal Society of London 60: 489–498.

4. Jackson DA, Somers KM (1991) The spectre of “spurious” correlations. Oecologia 86: 147–151. doi:10.1007/BF00317404.

5. Gelman A, Hill J (2007) Data Analysis Using Regression and Multilevel/Hierarchical Models. Cambridge University Press.
